# Demographic characteristics of free-roaming dogs (FRD) in rural and urban India following a photographic sight-resight survey

**DOI:** 10.1038/s41598-019-52992-y

**Published:** 2019-11-12

**Authors:** Harish Kumar Tiwari, Ian D. Robertson, Mark O’Dea, Abi Tamim Vanak

**Affiliations:** 1 College of Veterinary Medicine, School of Veterinary and Life Sciences, Murdoch University, Perth, Western Australia Australia; 2 Ashoka Trust for Research on Ecology and the Environment (ATREE), Bangalore, India; 3 AUSVET, 5 Shuffrey Street, Fremantle, Perth, Western Australia Australia; 4 China-Australia Joint Research and Training Center for Veterinary Epidemiology, Huazhong Agricultural University, Wuhan, 430070 Hubei China; 5 Wellcome Trust/DBT India-Alliance Fellow, Hyderabad, India; 6 School of Life Sciences, University of KwaZulu-Natal, Durban, South Africa

**Keywords:** Animal behaviour, Population dynamics

## Abstract

An understanding of the core demographic characteristics of the sub-populations of FRD is essential to effectively implement both rabies control interventions through mass vaccination of FRD, and dog population control programmes. This study compares the data obtained following photographic sight-resight surveys in rural (Shirsuphal village in west India) and urban (Municipal Corporation Panchkula in north India) locations . A total of 263 and 1408  FRD were seen at least once through 617 and 3465 sightings in the rural and urban sites, respectively. The rural location had a lower proportion of females (OR 0.5, 95% CI 0.4–0.7) and a higher proportion of poor and fair conditioned dogs (OR 1.8, 95% CI 1.3–2.3) compared to the urban setting. The rural site also had fewer active FRD (OR 0.6, 95% CI 0.5–0.7) and FRD were less likely to be sighted within 20 m of garbage points (OR 0.3, 95% CI 0.2–0.3) compared to the urban site. The demographic composition of the FRD population was found to vary within the urban location, with the odds of sighting a de-sexed dog being significantly higher in residential areas compared to other areas. The study underlines the importance of knowing the demographic composition of FRD for implementation of effective interventions against rabies. Fewer female dogs in the rural location indicate that spaying could be an effective tool for dog population management in this setting, while presence of dogs within 20 m of garbage points in urban settings highlights that an improved garbage management may reduce the carrying capacity of the urban locality resulting in smaller FRD population. It is concluded that quick and low cost surveys can generate useful demographic data for FRD in urban and rural settings which can be useful to understand the epidemiology of rabies and its control.

## Introduction

Free-roaming dogs (FRD) pose a serious threat to human health in countries where dog-bite related rabies is endemic, as well as causing environmental contamination with faeces, spreading garbage waste, damage to property and noise pollution^1–3^. The epidemiology of human rabies is intrinsically connected with the presence of rabies virus in FRD, and thus understanding the ecology of these dogs is imperative when developing and implementing control programmes for rabies, as well as other zoonotic diseases^4–8^. The structure and turnover of the FRD population is based on the characteristics of the dog’s demography including gender and age ratios, body condition, birth rates, success of rearing, mortality and survival rates^9^.

The presence of FRD on the streets of urban and rural areas in rabies endemic countries is maintained by a combination of factors, namely indiscriminate breeding of unowned dogs, unrestricted movement of semi-owned dogs and abandonment of owned dogs by irresponsible owners^10^. The World Health Organisation (WHO) recommends control of rabies through annual mass vaccination of FRD, with coverage of at least 70% of the population required to break the disease’s transmission cycle^11^. This percentage accounts for the loss of herd immunity levels resulting from the turnover of the dog population due to deaths, births and migrations^12^. Knowledge of the core demographic characteristics of sub-populations of FRD, such as male-female ratios, age composition of the population, social behaviour with respect to their dependence on edible litter/garbage and their activity level, is important to effectively implement both rabies control interventions and dog population control programmes^13^.

Animal Birth Control programmes have been implemented in some urban localities in India, although at many places the efforts have been irregular and sporadic^14,15^. These efforts are often implemented without considering the demographic composition of the FRD in the area of application, resulting in little or no reduction in the population. Furthermore, there are few epidemiological studies on the demographic composition of FRD in India, where rabies is endemic and the majority of human mortality from rabies is linked to dog bites^8^. Although some studies have been conducted on the demographics of FRD in Eastern India (West Bengal) in urban settings^16,17^, few studies from rural areas have been undertaken^18^. In this study we present the demographic details of FRD in Shirsuphal village in western India (rural location), and compare these with various residential and industrial sectors of the urban municipality of Panchkula in north India, through a series of photographic capture-recapture surveys of individually identifiable FRD undertaken on 5–7 occasions on predetermined tracks. We also discuss the various factors that possibly influence the FRD demography in rural and urban settings and the implications of such data for implementing effective rabies control interventions.

## Results

### Sighting variability and the demographic characteristics of free roaming dogs

At the rural site a total of 263 distinct dogs were identified through 617 sightings during the seven surveys conducted over the nine-day survey period. The demographic details of the FRD seen at least once are presented in Table 1. Variations in the number of dogs sighted on each survey, along with meteorological data, are presented in the Supplementary Table S1. The number of dogs sighted each day ranged from 106 to 52 with a declining trend as the survey progressed. This decrease followed a linear relationship with a negative slope (R^2^ = 0.63, *y* = −6.57*x* + 114.43). The number of active FRD during the survey period remained similar across survey days but a significant variation was observed in the number of FRD with respect to their proximity (≤20 m) to garbage points (Table 2).

**Table 1:** Demographic details (gender, age distribution and body condition) of the free roaming dogs sighted on each survey occasion during the enumeration survey (7 occasions) in the rural survey (Shirsuphal village, Baramati, Pune).

Survey occasion	Number of dogs sighted	Gender*		Age^@^				Body condition^@^		
		Male (%)	Female (%)	Pup (%)	Young (%)	Adult (%)	Old (%)	Good (%)	Fair (%)	Poor (%)
1	93	56 (62)	34 (38)	8 (9)	17 (18)	63 (68)	5 (5)	57 (61)	22 (24)	14 (15)
2	106	70 (75)	23 (25)	11 (10)	11 (10)	74 (70)	10 (9)	54 (51)	41 (39)	10 (10)
3	103	67 (68)	31 (32)	8 (8)	21 (20)	63 (61)	11 (11)	56 (54)	35 (34)	13 (13)
4	91	66 (78)	19 (22)	2 (2)	18 (20)	64 (70)	7 (8)	53 (58)	31 (34)	7 (8)
5	90	58 (68)	27 (32)	9 (10)	17 (19)	54 (60)	10 (11)	39 (43)	37 (41)	15 (16)
6	82	45 (60)	30 (40)	4 (5)	11 (13)	59 (72)	8 (10)	53 (62)	27 (32)	5 (6)
7	52	33 (65)	18 (35)	2 (4)	10 (19)	35 (67)	5 (10)	43 (83)	8 (15)	1 (2)
		170# (71)	70 (29)	17 (6)	34 (13)	197 (75)	15 (6)	143 (54)	89 (34)	31 (12)
Test for independence over the seven surveys		χ² = 10.33, p = 0.17		χ² = 23.7, p = 0.31				χ² = 32.8, p = 0.003		

Rows indicate the number of confirmed unique animals in the relevant category; *Gender of 23 FRD could not be verified; ^@^Age and Body condition were assessed based on visual characteristics as: Pup (<6 months), Young (6 months to 1 year), Adult (≥1 to 7 years), and Old (>7 years); Good, Fair and Poor, respectively.

**Table 2:** Details of the activity and sightings within 20 m of garbage of free roaming dogs sighted on each survey occasion during the rural survey (Shirsuphal village, Baramati, India).

Survey occasion	Number of dogs sighted	Activity (%)		Proximity to garbage (%)	
		Active	Not active	≤20 m	>20 m
1	93	40 (43)	53 (57)	32 (34)	61 (66)
2	106	42 (39)	64 (61)	34 (32)	72 (68)
3	103	42 (41)	61 (59)	25 (24)	78 (76)
4	91	36 (40)	55 (60)	15 (16)	73 (84)
5	90	41 (46)	49 (54)	17 (19)	73 (81)
6	82	40 (49)	42 (51)	16 (20)	66 (80)
7	52	22 (42)	30 (58)	9 (17)	43 (83)
Total	617	263 (43)	354 (57)	148 (24)	469 (76)
Test for independence over the seven surveys		χ² = 1.004, p = 0.99		χ² = 15.98, p = 0.025	

At the urban site a total of 1408 unique FRD were identified through 3465 reliable sightings in the 14 sectors of Panchkula Municipal Corporation administrated areas during September-October 2016. The demographic details of these FRD are displayed in Table 3. The details of the number of FRD sighted each day, along with the meteorological details for the urban survey, are presented in the Supplementary Table S2.

**Table 3:** Demographic details (gender, age distribution, neutering status and body condition), their respective distribution for the FRD sighted in the different sectors of the urban location (Panchkula Municipal Corporation administrated sectors).

Sector	Unique dogs identified	Gender^$^		Age^†^				Neutered		Body condition^†^		
		Male (%)	Female (%)	Pup (%)	Young (%)	Adult (%)	Old (%)	Yes (%)	No (%)	Good (%)	Fair (%)	Poor (%)
1&5	148	82 (55)	66 (45)	13 (9)	1 (1)	134 (90)	0	11 (7)	137 (93)	123 (83)	18 (12)	7 (5)
2	127	57 (51)	55 (49)	13 (10)	12 (9)	94 (75)	8 (6)	45 (35)	82 (65)	89 (70)	25 (20)	13 (10)
6	85	51 (61)	32 (39)	5 (6)	11 (13)	69 (79)	2 (2)	10 (12)	75 (88)	63 (74)	17 (20)	5 (6)
7	69	40 (58)	29 (42)	11 (16)	17 (25)	40 (58)	1 (1)	29 (42)	40 (58)	58 (84)	7 (10)	4 (6)
8	112	54 (55)	44 (45)	15 (13)	8 (7)	81 (73)	8 (7)	18 (16)	94 (84)	70 (62)	31 (28)	11 (10)
9	97	54 (57)	40 (43)	3 (3)	14 (14)	67 (70)	12 (13)	31 (32)	66 (68)	58 (60)	29 (30)	10 (10)
12	86	42 (52)	39 (48)	3 (3)	4 (5)	74 (86)	5 (6)	32 (37)	54 (63)	65 (75)	13 (15)	8 (10)
16	114	65 (61)	42 (39)	8 (7)	13 (11)	91 (80)	2 (2)	36 (31)	78 (69)	79 (69)	18 (16)	17 (15)
17	60	39 (65)	21 (35)	2 (3)	8 (13)	49 (82)	1 (2)	16 (27)	44 (73)	45 (75)	11 (18)	4 (7)
18	92	59 (64)	33 (36)	5 (5)	25 (27)	56 (61)	6 (7)	17 (18)	75 (82)	71 (77)	3 (3)	18 (20)
8 (P)^	37	22 (59)	15 (41)	3 (8)	0	33 (89)	1 (3)	6 (16)	31 (84)	29 (78)	8 (22)	0
IAP 1*	168	86 (55)	69 (45)	12 (6)	17 (10)	135 (82)	4 (2)	44 (26)	124 (74)	90 (53)	55 (33)	23 (14)
IAP 2#	144	74 (57)	55 (33)	15 (11)	5 (3)	118 (82)	5 (4)	26 (18)	118 (82)	76 (53)	40 (28)	28 (19)
BP,IC,RC@	69	37 (56)	29 (44)	1 (1)	7 (11)	61 (88)	0	20 (29)	49 (71)	39 (56)	3 (4)	27 (40)
	**1408**	**762 (57)**	**569 (43)**	**109 (8)**	**142 (11)**	**1102 (77)**	**55 (4)**	**330 (23)**	**1078 (77)**	**955 (68)**	**278 (20)**	**175 (12)**
Test for independence over fourteen survey tracks		χ^2^ = 7.9, p = 0.8		χ^2^ = 146.2, p < 0.001				χ^2^ = 76.3, p < 0.001		χ^2^ = 160.6, p < 0.001		

^Sector 8 perimeter, *Industrial Area Part 1, ^#^Industrial Area Part 2, ^@^Budhanpur, Indira Colony, Rajeev Colony; ^$^Gender of 77 FRD could not be verified; ^†^Age and body condition were recorded by visual appreciation as: Pup (<6 months), Young (6 months to 1 year), Adult (≥1 to 7 years), and Old (>7 years); and Good, Fair and Poor, respectively.

The proportion of dogs sighted (of the total unique FRD identified in each sector of Panchkula) on each day of the survey varied from 21 to 67%. The proportion of animals sighted on each day of the survey mostly followed a linear relationship with a positive slope, except for the residential sectors (9, 12, 16 and 17), where the trend was negative. Wind velocity and ambient temperature during the survey had a negative correlation (r = −0.3, p = 0.01; r = −0.5, p = 0.001, respectively) with the number of dogs sighted. Although no significant variation was observed in the gender composition of the FRD (χ^2^ = 7.9, df = 13, p = 0.8), a significant variation was found in the age and body condition composition of the FRD observed in each of the surveyed sectors (χ^2^ = 146.19, df = 39, p < 0.001 and χ^2^ = 160.6, df = 26, p < 0.001, respectively).

The number of dogs observed as active in Panchkula was negatively correlated with the ambient temperature at the time of the survey (r = −0.4, p = 0.000056) and varied significantly across the surveyed sectors (χ^2^ = 27.6, df = 13, p = 0.01). Similarly, the number of dogs sighted within 20 meters of garbage dumps/accumulated litter differed significantly between the sectors (χ^2^ = 287.5, df = 13, p < 0.001). Of the total 3465 sightings of FRD in all urban sectors, 1884 (54%) involved animals that were active and 1678 (48%) were sightings of dogs within 20 m of garbage points/dump sites (Table 4).

**Table 4:** Details of activity and sightings within 20 m of a garbage point for free roaming dogs sighted across 14 sectors of the urban location (Panchkula Municipal Corporation administrated area).

Sector	Sightings (n)	Activity		Proximity to garbage points	
		Active (%)	Not-active (%)	≤20 m (%)	>20 m (%)
1&5	308	181 (59)	127 (41)	175 (57)	133 (43)
2	313	163 (52)	150 (48)	105 (34)	208 (66)
6	164	93 (57)	71 (43)	73 (45)	91 (55)
7	192	107 (56)	85 (44)	29 (15)	163 (85)
8	250	109 (44)	141 (56)	61 (24)	189 (76)
9	280	119 (43)	161 (58)	94 (34)	186 (66)
12	199	111 (56)	88 (44)	70 (35)	129 (65)
16	277	128 (46)	149 (54)	135 (49)	142 (51)
17	136	85 (63)	51 (38)	12 (9)	124 (91)
18	183	121 (66)	62 (34)	36 (20)	147 (80)
8(P)^	120	59 (49)	61 (51)	70 (58)	50 (42)
IAP* 1	452	223 (49)	229 (51)	433 (96)	19 (4)
IAP* 2	451	278 (62)	173 (38)	285 (63)	166 (37)
BP,IC,RC#	140	107 (76)	33 (24)	100 (71)	40 (29)
**Total**	**3465**	**1884** (54)	**1581** (46)	**1678** (48)	**1787** (52)
Test for independence over fourteen survey tracks		χ^2^ = 27.6, p = 0.01		χ^2^ = 287.5, p < 0.001	

^Sector 8 perimeter, *IAP = Industrial Area Part, ^#^Budhanpur, Indira Colony, Rajeev Colony.

**Figure 1:**
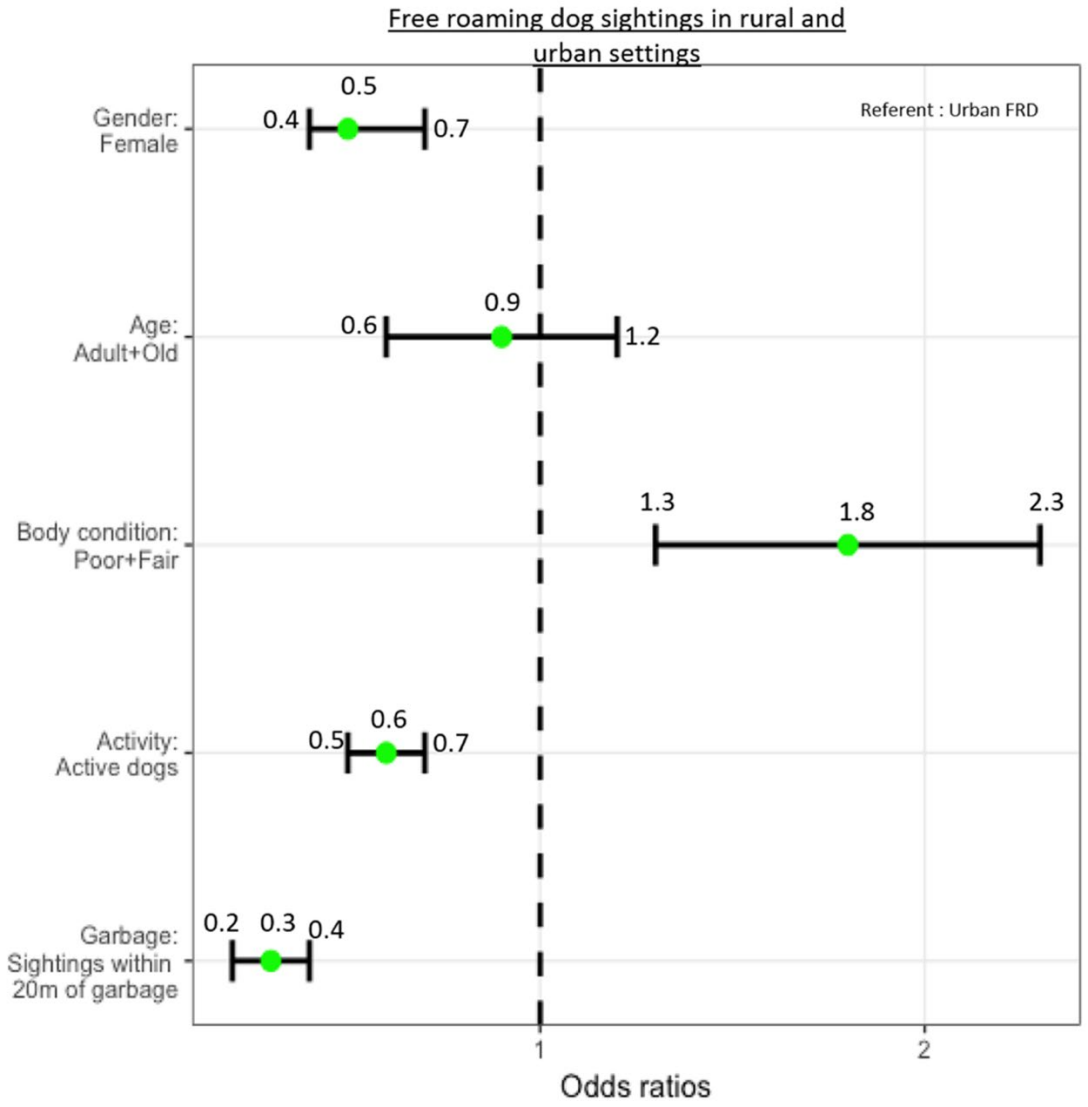
Graphical representation of the likely sightings of FRD according to gender, age, body condition, activity level and proximity to garbage (≤20 m) in the rural (Shirsuphal village) and urban (sectors of Panchkula Municipal Corporation) study sites during the enumeration survey carried out in September-October 2016. *The dots represent the odds ratio and the bars represent the 95% confidence limits.

The comparison of the demographic characteristics of rural and urban FRD is presented in Supplementary Table S3. The odds of sighting a female dog were significantly lower in the rural area (Shirsuphal) compared to the urban area (Panchkula) (OR 0.5, 95% CI 0.4–0.7, p < 0.001). The likelihood of sighting a FRD with a poor or fair body condition was higher in the rural setting compared to the urban setting (OR 1.8, 95% CI 1.3–2.3, p < 0.001). Rural FRD were less active (OR 0.6, 95% CI 0.5–0.7, p < 0.001) and less likely to be sighted within 20 m of a garbage point (OR 0.3, 95% CI 0.2–0.3, p < 0.001) than urban FRD (Fig. 1).

### Variations in the composition of the free-roaming dog population within the urban region

**Figure 2:**
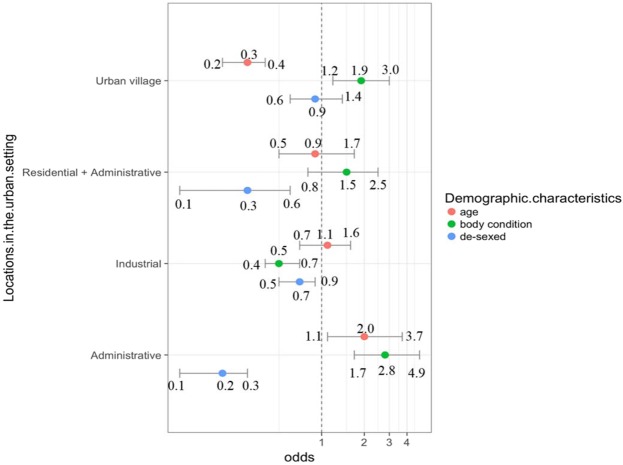
Graphical representation of the likely sightings of FRD (odds ratios and their 95% confidence intervals) according to age, body condition and de-sexing status during the enumeration survey carried out in the residential, industrial, administrative and mixed sectors of Panchkula Municipal Corporation during September-October 2016*. *The dots represent the odds ratio and the bars the 95% confidence limits of the odds.

Comparison of the composition of the FRD population observed in different localities during the urban survey is presented in Supplementary Table S4. The proportion of adult and old dogs in the administrative sector was significantly higher (OR 2.0, 95% CI 1.1–3.7, p = 0.01) than that for FRD in the residential sectors. In contrast the proportion of adult and old dogs in the organised sectors in the urban location was significantly lower (OR 0.3, 95% CI 0.2–0.4, p < 0.001) than the urban residential sectors. The proportion of the FRD with a good body condition was significantly higher in the administrative areas (OR 2.8, 95% CI 1.7–4.9, p = 0.01), and the urban village (OR 1.9, 95% CI 1.2–3.0, p < 0.001), but lower in the industrial sectors (OR 0.5, 95% CI 0.4–0.7, p < 0.001) compared to the residential sectors. The residential areas had a significantly higher proportion of de-sexed FRD (ear-notched) compared with the industrial, administrative, and the part residential, part administrative sectors (Fig. 2).

## Discussion

A series of photographic sight-resight surveys of individually identifiable FRD was conducted in an urban and rural setting in India to estimate the population size of FRD and the data obtained were analysed to describe the demographic characteristics of FRD to further our understanding of the implications of the demographics of FRD in intervention programmes against rabies or dog population management measures.

Although, the enumeration surveys were conducted at different times of the year, and only one village could be included from each location due to resource constraints, the methodology followed was consistent between surveys and each survey covered the entire selected village or sector. Nonetheless, there could be an inherent difference in the level of detectability of urban and rural FRD which may be a potential limitation of this study. Further, only one survey could be undertaken on the perimeter area separating the residential sectors in the urban location due to resource constraints. It is possible that dogs that were sighted in that perimeter area were in transit and may have actually resided in one of the neighbouring sectors, but we could not confirm this. The influence of extrinsic factors and demographic composition of FRD in urban and rural locations is described in the following sections.

### Influence of temperature on FRD sightings

The ambient temperature of the survey day was found to have a significant negative effect on the number of FRD sighted, an observation that was more pronounced in the urban survey, where a larger spatial and temporal dataset were available. Similar results were reported in a study in Berkeley, USA by Berman and Dunbar^19^, where the sightings of FRD declined when temperatures rose above 24 °C. We observed higher counts in morning sessions that were cooler (26 °C on average), compared to the warmer (32 °C average) afternoon sessions. Increased number of sightings during the cooler periods of the day is likely associated with increased movements of the dogs to seek food or company. In contrast with increasing temperatures dogs would seek shaded or cooler shelters resulting in less frequent sightings on the roads. Similar findings were also reported in West Bengal, India by Oppenheimer and Oppenheimer^17^. The influence of overcast conditions and rainfall on the sightings of FRD in the rural location is discussed in more detail in Tiwari, *et al*.^1^.

### Gender ratio

The gender ratio in Shirsuphal village (rural) was heavily skewed in favour of males (1: 2.45) (71% males), which was similar to that reported from villages in the vicinity of the Great Indian Bustard Sanctuary in the neighbouring district of Solapur, as well as from Bangladesh, Chile, Indonesia, and the Republic of South Africa^12,18,20–22^. This disparity could be attributed to the preference of farming communities for male dogs^21,23^ or high female dog mortality^12,24^. In contrast a closer male-female parity was observed in the urban study (Panchkula) (1.34: 1) (57% males), which is consistent with estimates from other urban studies in West Bengal, India (1.37: 1) and Bhutan (1.31: 1)^16,25^. The variations in the gender ratios between different sectors (1.07–1.85: 1) in Panchkula could be attributed to the varying degree of human influence on FRD, which would be expected to be higher in residential sectors than in sectors that comprised open areas (parks, school playgrounds, markets, industrial areas)^26^. The ABC programme in Panchkula may have also contributed to gender parity in FRD^15^, but with only 23% of dogs identified as de-sexed (ear-notched), the impact would not be expected to fully explain the gender ratio in the urban location.

### Age composition of FRD

No significant difference was observed in the age composition of FRD in the rural and urban surveys, which was similar to findings reported in other countries^12,18,20,25,27,28^. The low percentage of pups and young dogs in both rural (17%) and urban (18%) settings could be due to high early mortality^12^. It may be argued that the time of the survey in rural Shirsuphal was an influencing factor for the infrequent sightings of puppies and young dogs because the survey was undertaken before (early June) the whelping season in September-October^16^, but a low percentage was also observed in urban Panchkula where the study was undertaken in September-October supporting the hypothesis of early-age mortality in FRD irrespective of the location. Human influences, such as motor-vehicle accidents and deliberate poisoning, also contribute to the death of FRD puppies^8,22,29,30^. The stress caused by biannual breeding in reproductive females has also been cited as a potential reason for early juvenile mortality^6,31^. Furthermore, the absence of any communal or group care of pups by other bitches witnessed in FRD (as opposed to wild canids) also contributes to the low survival of juveniles^32^. Butler and Bingham^6^, observed that extra nutritional pressure exerted on reproductive free-roaming bitches by the sympatric semi-owned dogs may also result in higher mortality of puppies. Others have reported that dogs after the age of 4 to 6 months may move to neighbouring villages with less competition for food/shelter resulting in a lower percentage of young dogs, a possibility which cannot be ruled out in the present study^33–35^. However, as the population size is driven by a large number of reproductively active animals coupled with large litter sizes^12,36^, a high proportion of reproductively active animals is an indicator of the high fecundity of the population in both the urban and rural survey sites.

### Body condition and the sightings near garbage points

The likelihood of sighting a FRD of poor or fair condition in rural Shirsuphal was significantly higher (OR 1.8) than in urban Panchkula. This may be related to the availability of food as FRD were less likely to be sighted within 20 m of garbage points in the rural study (OR 0.3) than in the urban study. Tenzin, *et al*.^37^, also reported a high proportion of FRD with a good body condition in Bhutan and attributed this to ready access to food and the local community’s responsibility to feed FRD. In this study, besides FRD in urban Panchkula sourcing feed from the garbage points, a higher percentage of urban (72%) than rural (39%) residents fed the FRD due to compassionate or religious reasons^38,39^. Although only 24% of the FRD were found within a 20 m radius of garbage dumps/sites in rural Shirsuphal, it is possible that they scavenge from such sites in the evenings^26,32,40,41^. The good body condition of more than half of the sighted FRD and a lack of congregation around garbage sources provides evidence that the FRD in Shirsuphal are not typical of feral dogs, but are more likely to have some level of human association and at least some of them could be loosely categorised as owned as it is highly unlikely for a FRD population to remain in a state of good health without some form of human intervention^42^.

Totton, *et al*.^43^, cited that ABC programmes were key drivers of better health conditions for the FRD in urban Jaipur, north India. The benefits of conducting sustained ABC programmes to improve the body condition of unowned dogs is also confirmed by the results of studies in Dhaka, Bangladesh and in urban Jodhpur, India^28,44^. Potentially the better body condition observed in the FRD in the urban survey could also be a result of the ongoing sterilisation programme in Panchkula, although only 23% of FRD dogs were identified as neutered (ear-notched).

### Activity

The sightings of FRD in urban Panchkula that were involved in some kind of activity, such as walking, running, foraging or playing, was significantly higher (OR 1.6) than that in rural Shirsuphal. The percentage of active FRD in urban settings (54%) was comparable to that observed in California, USA^19^ (56%). Although some studies suggest that the activity of FRD varies according to the time of the day^19,45^, in this study there was no apparent temporal pattern in their activity classification, although the proportion of FRD undergoing an activity was negatively correlated with the ambient temperature at the time of the survey.

In the urban survey at Panchkula most of the FRD categorised as “not-active” were sighted under parked cars, even when other places of shade were available. Although availability of food is considered the primary cause for the high number of FRD^46,47^, the presence of these “shelters” could also be an important factor contributing to the higher “carrying capacity” of an urban environment. Construction of dog-proof enclosed parking lots may contribute to the control of the FRD population in urban environs as they would deny the FRD temporary shelters - an essential component for their survival.

### Free-roaming dog demography and relationships to ABC programmes

The proportion of female dogs in Shirsuphal village was much lower than in urban Panchkula indicating that de-sexing of females is potentially an economically viable option for the control of the FRD population in this location. The same, however, may not be true in urban settings where there were comparable numbers of males and females, and as such de-sexing of both males and females may yield a faster population control in these locations. A salient finding of FRD in the urban settings was the significantly higher number of ear-notched (de-sexed) FRD in residential sectors than in other sectors (Fig. 2). This is most likely because the FRD are more easily accessible for capture in residential areas compared to other sectors. The process of neutering a FRD involves capture of the dog, a procedure which is very challenging and the dogs that are wary of human interaction often prove difficult, if not impossible, to catch. The challenges of catching a FRD to administer interventions is a serious impediment towards the control of rabies in canines^48,49^ and is a major cause for its persistence in countries, such as India, where the disease is endemic. Nevertheless, the efficacy of ABC measures in Panchkula remains doubtful in light of the wide disparity between the proportions of neutered FRD in the different sectors/areas. Demographic surveys, such as the one described in this study, thus also help assess the efficacy of ABC programmes.

The assumption that sterilisation can reduce the density of FRD resulting in fewer dog-bites and thus control the incidence of rabies^50^ can only be verified by studying the dynamics of population through a longitudinal observational study. In Panchkula, where ABC programmes are reportedly irregular and sporadic (Executive Officer, Municipal Corporation, Panchkula), it is likely that FRD do not live long as no aged dogs were sighted among the sterilised dogs. Reece and Chawla^51^, while discussing control of rabies in Jaipur, India, argued that sterilisation followed by vaccination against rabies results in life long immunity in stray dogs presumably due to their short lifespan, reported to average 2.6 years^16^. Nevertheless, the possible reduction in the density of the dogs due to sterilisation is likely to be mitigated by high population turnover and immigration of FRD from neighbouring sectors. Moreover, host density does not appear to affect the transmission dynamics for dog-related rabies due to a low reproductive number (R_0_) for the disease^52^.

In contrast, the dynamics of disease transmission depends largely on population size and the factors that sustain high numbers of the reservoir hosts^53^. Although mass vaccination of FRD has been widely recommended for the control of rabies^54,55^, this strategy has had very little success in India. This is likely due to the tendency of FRD to group around accumulated garbage which is enhanced when there is poor garbage management, which it turn makes it difficult for vaccine administrators to catch dogs for parenteral vaccination^56^. Besides inaccessibility of dogs for mass vaccination^57^, a factor that works against mass vaccination is the inability of malnourished or poor body conditioned FRD to sero-convert and sustain the population immunity at critical levels^42,58^. Such immune response constraints may not apply to areas such as Panchkula, where the majority of the FRD (68%) were found to be in good body condition. However, as cities improve their solid waste management, leading to a reduction of the resources that sustain dog populations in cities such as Panchkula, it may in the short-term create cohorts of under-nourished dogs.

We have demonstrated that quick and relatively low-cost surveys such as described in Tiwari, *et al*.^1^ and Tiwari, *et al*.^59^ can not only provide robust population estimates for FRD, but can also be used to generate useful demographic data for dogs in urban and rural areas of a rabies endemic country such as India. Such data provide useful insights into the various factors that need to be considered for understanding the epidemiology of rabies and its control.

## Materials and Methods

### Study area

- Rural: The rural surveys were conducted in the village of Shirsuphal in Baramati town of Pune District in Maharashtra state of western India (18°18′49.08″N and 74°34′44.40″E) from the 4^th^ to 13^th^ June 2016. The village has human settlements interspersed with farmland. Sixteen km of roads, of which 12 are bitumen, connect the various settlements. The village comprises of 1161 households (www.censusofindia.gov.in, as accessed in July 2016). The study excluded FRD on farmlands sighted at a distance of 200 meters or more from the roads. In the month of June, the average humidity in Baramati is 72% with an ambient temperature range of 23 °C to 32 °C (https://www.timeanddate.com/weather/india/baramati). The survey was conducted using the roads and trails along the human habitats that connect the various settlements^1^.
- Urban: The residential and industrial areas under the administrative control of the Municipal Corporation, Panchkula (30°38′58.58″N and 76°49′52.73″E) were surveyed during the months of September and October 2016. Panchkula is one of the highly urbanised and planned districts in India^60^ and the Municipal Corporation administered area is comprised of wards that are further divided into sectors. The number of sectors in each ward varies from 1 to 6, and includes highly organised residential, administrative and industrial sectors interspersed by unorganised slums and villages^61^. Fifteen sectors were selected through purposive (industrial, unorganised and mix sectors) and random (residential sectors) selection and included residential (sectors 2, 8, 9, 12, 16 and 17); administrative (sectors 1, 5); industrial (sectors IAP I and IAP II); and part–residential part-public areas such as hospitals, colleges and parks (sector 6). Two of the surveyed sectors (7 and 18) included an urban village where, although the survey route was bitumen, many alleys branched out into densely unorganised settlements (slums). Such alleys were not included in the survey due to the resistance of the residents to participation. However an unorganised area included in the Municipal Corporation limits, comprising three colonies (Budhanpur, Rajeev Colony, Indira Colony - referred to as BP, RC, IC, respectively throughout this paper), was surveyed. While the possibility of FRD moving from one sector to another could not be completely excluded, their movement was restricted within residential areas by solid brick walls surrounding the areas. The counts of FRD were undertaken in the perimeter area of sector 8 on the roads connecting the residential sectors. In contrast the industrial areas did not have such defined walled restrictions. The survey was conducted inside each sector along the connecting bitumen roads. The surveyed area comprised 6337 households (www.censusofindia.gov.in, as accessed in July 2016). The average temperature observed in September/October 2016 was 29 °C/26 °C with an average humidity of 75%/69%, respectively (www.timeanddate.com/weather/india/panchkula).

### Field methodology

A consistent methodology through a series of photographic sight-resight surveys of the FRD was followed in the rural (Shirsuphal village) and urban (sectors under administrative control of Panchkula Municipal Corporation) locations (Tiwari, *et al*.^1^ and^59^). Briefly, the selected areas were traversed by a team of two observers riding a motorcycle at a constant speed of ~20 Km/hour following a predetermined route (survey-track) on alternate mornings and evenings/afternoons for five to seven occasions. The survey-tracks and the teams remained unchanged throughout the survey period for a particular survey track and were equipped with a GPS device (Garmin eTrex20 GPS device, www.garmin.com), a digital camera (Nikon COOLPIXA900) and writing materials. The surveys were conducted during mornings (6.30–8.30 for the rural and 6.00–8.00 for the urban locations) and evenings/afternoons (17.00–19.00 for the rural and 16.00–18.00 for the urban locations), except for the three unorganised colonies (BP, RC and IC) in the urban settings where the residents resisted the survey during the afternoon sessions. The duration of the surveys were constant for a particular survey track and lasted for 1 to 2 hours, depending on the track length and number of FRD sightings on the day of the survey. The surveys covered all bitumen routes in the selected areas that were frequented by human and FRD alike^62^ and covered the entire village/sector. The lengths of the two survey tracks in Shirsuphal were 7.5 and 6 km, while those in the urban location of Panchkula varied from 4.2 to 14.7 km (Supplementary Table S3).

Each FRD sighted during the survey was photographed and details recorded on its gender (male/female/not verified), age (pups/young/adult/old), body condition (poor/fair/good), reproductive status (pregnant/in-oestrous/lactating/notched), and details of the coat colour (single coloured/bicoloured/tricoloured/mixed/striped) along with its location (GPS waypoints). The photographs of all FRD were matched with details on the datasheet after each survey to ascertain if a FRD had been seen for the first time or if it was a resighted one. A list of FRD sighted at least once during the entire survey period for each location was compiled and used for analysing the attributes of the dogs.

Besides the assigned sites of garbage disposals (garbage dumps/points), many temporary accumulations of litter along the roads were witnessed, especially in the rural settings. The proximity of the FRD to such garbage dumps/points or accumulated litter (presence ≤20 m of such sites), the activity of the FRD at the time of the survey (active/inactive), the reproductive status of adult female dogs (if lactating or in oestrus), and their de-sexed status (left ear-notched indicating de-sexed/not notched indicating entire) were also recorded. A FRD was recorded as active if it was found walking, running, playing or foraging and as inactive if observed sitting, lying or sleeping.

### Data entry and analysis

All population survey data were entered and organised in a spreadsheet (Microsoft Excel 2013, Redmond, USA) after each survey. Every dog sighted during the complete survey was marked as ‘1’ or ‘0’ as having been ‘sighted’ or ‘not sighted’ on a particular survey. Chi-square tests for independence were used to examine variation in the counts of different categories (gender, age groups, body condition score, proximity to garbage) observed between the surveys. Regression analyses and χ^2^ tests were performed in R Programming Environment^63^. The R package “epitools” was used to calculate the odd ratios^64^.

### Ethical approval

Ethics approval for the observation of the FRD in the rural and urban areas was obtained from ATREE (Ashoka Trust for Research in Ecology and the Environment) (AAEC/101/2016).

## Supplementary information

Supplementary Tables
