# Author Correction: Demographic characteristics of free-roaming dogs (FRD) in rural and urban India following a photographic sight-resight survey

**DOI:** 10.1038/s41598-020-58147-8

**Published:** 2020-02-25

**Authors:** Harish Kumar Tiwari, Ian D. Robertson, Mark O’Dea, Abi Tamim Vanak

**Affiliations:** 1 College of Veterinary Medicine, School of Veterinary and Life Sciences, Murdoch University, Perth, Western Australia Australia; 2 Ashoka Trust for Research on Ecology and the Environment (ATREE), Bangalore, India; 3 AUSVET, 5 Shuffrey Street, Fremantle, Perth, Western Australia Australia; 4 China-Australia Joint Research and Training Center for Veterinary Epidemiology, Huazhong Agricultural University, Wuhan, 430070 Hubei China; 5 Wellcome Trust/DBT India-Alliance Fellow, Hyderabad, India; 6 School of Life Sciences, University of KwaZulu-Natal, Durban, South Africa

Correction to: *Scientific Reports* 10.1038/s41598-019-52992-y, published online 12 November 2019

This Article contains errors in Reference 62 which was incorrectly given as:

Elly, H. & Lex, H. Direct observation of dog density and composition during street counts as a resource efficient method of measuring variation in roaming dog population over time and between locations. *Animals* 7, 10.3390/ani7080057 (2017).

The correct reference is listed below as Reference 1:
