# Supplementary material for: Demographic characteristics of free-roaming dogs (FRD) in rural and urban India following a photographic sight-resight survey
Source: Sci Rep. 2019 Nov 12;9:16562. doi: 10.1038/s41598-019-52992-y (PMC6851138; doi:10.1038/s41598-019-52992-y)
Supplement: Supplementary file 1 — Supplementary Tables [file 41598_2019_52992_MOESM1_ESM.docx]

**Demographic characteristics of free-roaming dogs (FRD) in rural and urban India following a photographic sight-resight survey**

**(Running title: Free-roaming dog demography)**

*Harish Kumar Tiwari*^1, 2, 3^, Ian D Robertson^1, 4^, Mark O’Dea^1^, Abi Tamim Vanak ^2,5,6^*

**1. School of Veterinary Medicine, Murdoch University, Western Australia, Australia.**

**2. Ashoka Trust for Research on Ecology and the Environment (ATREE), Bangalore, India.**

**3. AUSVET, 5 Shuffrey Street, Fremantle, Perth, Western Australia, Australia.**

**4. China-Australia Joint Research and Training Center for Veterinary Epidemiology, Huazhong Agricultural University, Wuhan 430070, Hubei, China.**

**5. Wellcome Trust/DBT India-Alliance Fellow, Hyderabad, India.**

**6. School of Life Sciences, University of KwaZulu-Natal, Durban, South Africa.**

**Supplementary tables**

Supplementary Table S1 Details of number of Free-roaming dogs sighted during the sight-resight surveys carried in Shirsuphal village and fourteen survey tracks of Panchkula Municipal Corporation along with the meteorological data for each survey session.

| **Survey track** | **Total sightings** | **Unique dogs sighted** | **Date** | **Session** | **Count each day** | **Temp (^0^C)** | **Humidity**  **(%)** | **Wind velocity**  **(km/hr)** | **Climate** |
| --- | --- | --- | --- | --- | --- | --- | --- | --- | --- |
| **Rural (Shirsuphal)** | | | | | | | | | |
| A & B | 617 | 263 | 5/06/2016 | E | 93 | 32 | 55 | 7 | Sunny |
|  |  |  | 6/06/2016 | M | 106 | 26 | 80 | 2 | Overcast |
|  |  |  | 7/06/2016 | E | 103 | 32 | 55 | 6 | Overcast |
|  |  |  | 8/06/2016 | M | 91 | 27 | 78 | 6 | Overcast |
|  |  |  | 9/06/2016 | E | 90 | 35 | 42 | 4 | passing clouds |
|  |  |  | 12/06/2016 | E | 82 | 30 | 59 | 13 | passing clouds |
|  |  |  | 13/06/2016 | M | 52 | 30 | 70 | 19 | passing clouds |
| **Urban (Panchkula)** | | | | | | | | | |
| 8 | 250 | 112 | 16/09/2016 | M | 53 | 26 | 85 | 4 | Clear |
|  |  |  | 18/09/2016 | A | 44 | 34 | 57 | no wind | passing clouds |
|  |  |  | 21/09/2016 | M | 58 | 28 | 88 | no wind | Sunny |
|  |  |  | 22/09/2016 | A | 37 | 29 | 67 | 4 | passing clouds |
|  |  |  | 24/09/2016 | M | 58 | 26 | 82 | 4 | Sunny |
| 2 | 313 | 127 | 6/10/2016 | M | 58 | 24 | 85 | no wind | Clear |
|  |  |  | 6/10/2016 | A | 55 | 32 | 56 | no wind | Sunny |
|  |  |  | 7/10/2016 | M | 75 | 25 | 85 | no wind | Fog |
|  |  |  | 7/10/2016 | A | 61 | 31 | 61 | no wind | Sunny |
|  |  |  | 8/10/2016 | M | 64 | 24 | 85 | no wind | Fog |
| 12 | 199 | 86 | 12/10/2016 | A | 36 | 31 | 45 | 2 | Sunny |
|  |  |  | 13/10/2016 | M | 52 | 22 | 71 | no wind | Clear |
|  |  |  | 14/10/2016 | M | 51 | 21 | 72 | no wind | clear |
|  |  |  | 14/10/2016 | A | 33 | 30 | 44 | 4 | Sunny |
|  |  |  | 15/10/2016 | A | 27 | 30 | 43 | 2 | Sunny |
| IAP 1 | 452 | 168 | 3/10/2016 | M | 64 | 28 | 88 | no wind | Fog |
|  |  |  | 3/10/2016 | A | 71 | 33 | 63 | no wind | Haze |
|  |  |  | 4/10/2016 | A | 76 | 32 | 68 | no wind | Fog |
|  |  |  | 5/10/2016 | M | 83 | 27 | 79 | no wind | Fog |
|  |  |  | 5/10/2016 | A | 85 | 32 | 66 | 4 | Scattered clouds |
|  |  |  | 6/10/2016 | M | 73 | 24 | 85 | no wind | Clear |
| IAP 2 | 451 | 144 | 8/10/2016 | M | 68 | 24 | 85 | no wind | Fog |
|  |  |  | 9/10/2016 | M | 65 | 23 | 82 | no wind | Fog |
|  |  |  | 9/10/2016 | A | 76 | 31 | 53 | no wind | Sunny |
|  |  |  | 10/10/2016 | M | 88 | 24 | 78 | no wind | Scattered clouds |
|  |  |  | 10/10/2016 | A | 68 | 31 | 53 | no wind | Clear |
|  |  |  | 11/10/2016 | M | 86 | 22 | 82 | no wind | Fog |
| BP,IC,RC | 140 | 69 | 21/09/2016 | M | 29 | 28 | 88 | no wind | Sunny |
|  |  |  | 22/09/2016 | M | 26 | 25 | 82 | 7 | Partly sunny |
|  |  |  | 23/09/2016 | M | 32 | 24 | 85 | no wind | Clear |
|  |  |  | 24/09/2016 | M | 29 | 26 | 82 | 4 | Sunny |
|  |  |  | 25/09/2016 | M | 24 | 26 | 87 | 4 | Sunny |
| 9 | 280 | 97 | 15/09/2016 | M | 65 | 26 | 84 | no wind | Sunny |
|  |  |  | 15/09/2016 | A | 30 | 32 | 63 | no wind | passing clouds |
|  |  |  | 16/09/2016 | A | 46 | 33 | 58 | 2 | sunny |
|  |  |  | 18/09/2016 | M | 58 | 27 | 85 | no wind | Fog |
|  |  |  | 20/09/2016 | M | 57 | 27 | 89 | no wind | Fog |
|  |  |  | 20/09/2016 | A | 24 | 33 | 74 | no wind | passing clouds |
| 17 | 136 | 60 | 16/09/2016 | M | 25 | 26 | 85 | 4 | Clear |
|  |  |  | 16/09/2016 | A | 21 | 33 | 58 | 2 | sunny |
|  |  |  | 17/09/2016 | M | 28 | 27 | 87 | no wind | Sunny |
|  |  |  | 17/09/2016 | A | 18 | 33 | 62 | 2 | Scattered clouds |
|  |  |  | 18/09/2016 | M | 26 | 26 | 85 | 6 | passing clouds |
|  |  |  | 18/09/2016 | A | 18 | 34 | 57 | no wind | passing clouds |
| 16 | 277 | 114 | 25/09/2016 | M | 60 | 27 | 89 | no wind | passing clouds |
|  |  |  | 25/09/2016 | A | 33 | 31 | 64 | 2 | passing clouds |
|  |  |  | 26/09/2016 | M | 54 | 26 | 87 | 4 | Fog |
|  |  |  | 27/09/2016 | M | 57 | 26 | 85 | no wind | Fog |
|  |  |  | 30/09/2016 | A | 38 | 33 | 64 | no wind | Sunny |
|  |  |  | 1/10/2016 | A | 35 | 33 | 69 | no wind | Sunny |
|  |  |  |  |  |  |  |  |  |  |
| 1,5 | 308 | 148 | 12/09/2016 | M | 63 | 32 | 58 | 4 | Sunny |
|  |  |  | 14/09/2016 | A | 49 | 32 | 64 | 4 | passing clouds |
|  |  |  | 15/09/2016 | A | 63 | 32 | 63 | no wind | passing clouds |
|  |  |  | 16/09/2016 | M | 77 | 26 | 88 | no wind | Clear |
|  |  |  | 16/09/2016 | A | 56 | 26 | 85 | 4 | Clear |
| 8 (P) | 120 | 37 | 8/09/2016 | M | 18 | 28 | 87 | no wind | Clear |
|  |  |  | 8/09/2016 | A | 22 | 30 | 78 | no wind | Clear |
|  |  |  | 9/09/2016 | M | 19 | 28 | 85 | no wind | passing clouds |
|  |  |  | 10/09/2016 | M | 17 | 26 | 65 | 2 | Sunny |
|  |  |  | 12/09/2016 | A | 20 | 32 | 58 | 4 | Sunny |
|  |  |  | 15/09/2016 | A | 24 | 32 | 63 | no wind | passing clouds |
| 18 | 183 | 92 | 10/09/2016 | M | 31 | 28 | 73 | 4 | passing clouds |
|  |  |  | 10/09/2016 | A | 28 | 33 | 59 | 2 | Sunny |
|  |  |  | 11/09/2016 | M | 46 | 28 | 70 | no wind | Clear |
|  |  |  | 11/09/2016 | A | 53 | 33 | 58 | 6 | passing clouds |
|  |  |  | 12/09/2016 | M | 25 | 32 | 58 | 4 | Sunny |
| 6 | 164 | 85 | 9/09/2016 | M | 26 | 28 | 85 | no wind | passing clouds |
|  |  |  | 10/09/2016 | M | 40 | 28 | 73 | 4 | passing clouds |
|  |  |  | 10/09/2016 | A | 18 | 33 | 59 | 2 | Sunny |
|  |  |  | 11/09/2016 | M | 33 | 28 | 70 | no wind | Clear |
|  |  |  | 13/09/2016 | A | 19 | 29 | 64 | 4 | Partly sunny |
|  |  |  | 14/09/2016 | M | 28 | 32 | 64 | 4 | passing clouds |
| 7 | 192 | 69 | 7/09/2016 | A | 19 | 32 | 62 | 4 | passing clouds |
|  |  |  | 8/09/2016 | M | 32 | 28 | 87 | no wind | Clear |
|  |  |  | 8/09/2016 | A | 35 | 30 | 78 | no wind | Clear |
|  |  |  | 9/09/2016 | M | 40 | 28 | 85 | no wind | passing clouds |
|  |  |  | 10/09/2016 | M | 41 | 26 | 65 | 2 | Sunny |
|  |  |  | 11/09/2016 | A | 25 | 33 | 58 | 6 | passing clouds |

*†Session (A=Afternoon, E= Evening, M=Morning); ^ Temp= Temperature.*

**Industrial Area Part 1, # Industrial Area Part 2, @ Budhanpur, Indira Colony, Rajeev Colony, ^ Sector 8 perimeter*

Supplementary Table S2. Details of the track lengths, durations of photographic sight-resight survey, and population estimates of free roaming dogs during the enumerationsurvey carried out in Shirsuphal village and Panchkula Municipal Corporation administered sectors

| **Survey track** | **Date** | **Start time** | **End time** | **Survey track** | **Date** | **Start time** | **End time** |
| --- | --- | --- | --- | --- | --- | --- | --- |
|  |  |  |  |  |  |  |  |
| **Shirsuphal** | ***Population Estimate 391 ± 26** | | | | | | |
| **Track A** | **Track length: 7.5km** | | | **Track B** | **Track length: 6km** | | |
|  | 5/06/2016 | 0500pm | 0655pm |  | 5/06/2016 | 0500pm | 1841pm |
|  | 6/06/2016 | 0630am | 0814am |  | 6/06/2016 | 0630am | 0812am |
|  | 7/06/2016 | 0500pm | 0657pm |  | 7/06/2016 | 0500pm | 0700pm |
|  | 8/06/2016 | 0630am | 0810am |  | 8/06/2016 | 0630am | 0805am |
|  | 9/06/2016 | 0500pm | 0641pm |  | 9/06/2016 | 0500pm | 1842pm |
|  | 12/06/2016 | 0500pm | 0642pm |  | 12/06/2016 | 0500pm | 1843pm |
|  | 13/06/2016 | 0630am | 0758am |  | 13/06/2016 | 0630am | 0802am |
| **Panchkula** |  |  |  |  |  |  |  |
| **2** | **Track length: 13.8km; Population estimate 146 ± 8** | | | **8** | **Track length: 12.7km; Population estimate 14 ±11** | | |
|  | 6/10/2016 | 0605am | 0800am |  | 16/09/2016 | 0608am | 0811am |
|  | 6/10/2016 | 1612pm | 1810pm |  | 18/09/2016 | 0430pm | 0612pm |
|  | 7/10/2016 | 0615am | 0815am |  | 21/09/2016 | 0600am | 0750am |
|  | 7/10/2016 | 0419pm | 0615pm |  | 22/09/2016 | 0430pm | 0623pm |
|  | 8/10/2016 | 0626am | 0810am |  | 24/09/2016 | 0610am | 0810am |
| **6** | **Track length: 8.2km; Population estimate 140 ± 19** | | | **7** | **Track length: 6.7km; Population estimate 89 ± 8** | | |
|  | 9/09/2016 | 0622am | 0738am |  | 7/09/2016 | 0420pm | 0530pm |
|  | 10/09/2016 | 0630am | 0748am |  | 8/09/2016 | 0600am | 0736am |
|  | 10/09/2016 | 0410pm | 0542pm |  | 8/09/2016 | 0430pm | 0542pm |
|  | 11/09/2016 | 0605am | 0737am |  | 9/09/2016 | 0600am | 0746am |
|  | 13/09/2016 | 0412pm | 0545pm |  | 10/09/2016 | 0600am | 0736am |
|  | 14/09/2016 | 0606am | 0735am |  | 11/09/2016 | 0436pm | 0614am |
| **9** | **Track length: 8.4 km; Population estimate 114 ± 7** | | | **16** | **Track length:10.5km; Population estimate 142 ± 10** | | |
|  | 15/09/2016 | 0553am | 0826am |  | 25/09/2016 | 0620am | 0820am |
|  | 15/09/2016 | 0400pm | 0536pm |  | 25/09/2016 | 0435pm | 0614pm |
|  | 16/09/2016 | 0450pm | 0643pm |  | 26/09/2016 | 0610am | 0755am |
|  | 18/09/2016 | 0555am | 0805am |  | 27/09/2016 | 0620am | 0818am |
|  | 20/09/2016 | 0555am | 0800am |  | 30/09/2016 | 0436pm | 0614pm |
|  | 20/09/2016 | 0430pm | 0615pm |  | 1/10/2016 | 0426pm | 0612pm |
| **8 (P)** | **Track length: 5.8 km; Population estimate 44 ± 4** | | | **17** | **Track length: 5.7 km; Population estimate 78 ± 9** | | |
|  | 8/09/2016 | 0600am | 0700am |  | 16/09/2016 | 0558am | 0733am |
|  | 8/09/2016 | 0420pm | 0517pm |  | 16/09/2016 | 0432pm | 0535pm |
|  | 9/09/2016 | 0600am | 0700am |  | 17/09/2016 | 0602am | 0724am |
|  | 10/09/2016 | 0606am | 0702am |  | 17/09/2016 | 0438pm | 0539pm |
|  | 12/09/2016 | 0425pm | 0532pm |  | 18/09/2016 | 0601am | 0705am |
|  | 15/09/2016 | 0555am | 0653am |  | 18/09/2016 | 0440pm | 0543pm |
| **IAP 1** | **Track length:12.8km; Population estimate 190 ± 8** | | | **IAP 2** | **Track length: 9.0 km; Population estimate 164 ± 7** | | |
|  | 3/10/2016 | 0607am | 0806am |  | 8/10/2016 | 0406pm | 0548pm |
|  | 3/10/2016 | 0415pm | 0610pm |  | 9/10/2016 | 0610am | 0733am |
|  | 4/10/2016 | 0400pm | 0612pm |  | 9/10/2016 | 0419pm | 0610pm |
|  | 5/10/2016 | 0604am | 0812am |  | 10/10/2016 | 0606am | 0752am |
|  | 5/10/2016 | 0413pm | 0618pm |  | 10/10/2016 | 0412pm | 0545pm |
|  | 6/10/2016 | 0605am | 0819am |  | 11/10/2016 | 0620am | 0809am |
| **BP,IC,RC** | **Track length: 4.2 km; Population estimate 122 ± 9** | | | **1,5** | **Track length:14.7km; Population estimate 198 ± 15** | | |
|  | 21/09/2016 | 0559am | 0712am |  | 12/09/2016 | 0554am | 0758am |
|  | 22/09/2016 | 0610am | 0710am |  | 14/09/2016 | 0543am | 0659am |
|  | 23/09/2016 | 0602am | 0715am |  | 15/09/2016 | 0414pm | 0607pm |
|  | 24/09/2016 | 0553am | 0654am |  | 16/09/2016 | 0540am | 0804am |
|  | 25/09/2016 | 0559am | 0654am |  | 16/09/2016 | 0430pm | 0652pm |
| **12** | **Track length: 7.6 km; Population estimate 100 ± 7** | | | **18** | **Track length:10.4km; Population estimate 135 ± 15** | | |
|  | 12/10/2016 | 0411pm | 0526pm |  | 10/09/2016 | 0606am | 0803am |
|  | 13/10/2016 | 0640am | 0800am |  | 10/09/2016 | 0424pm | 0608pm |
|  | 14/10/2016 | 0624am | 0742am |  | 11/09/2016 | 0556am | 0801am |
|  | 14/10/2016 | 0430pm | 0538pm |  | 11/09/2016 | 0430pm | 0600pm |
|  | 15/10/2016 | 0418pm | 0524pm |  | 12/09/2016 | 0604am | 0815am |

Supplementary Table S3. A comparative analyses of the characteristics of free-roaming dogs from the rural (Shirsuphal village) and urban (Panchkula Municipal Corporation administrated area) locations presented as odd ratios (OR) and their 95% confidence intervals (CI)

| **Demographic Characteristic** | **Rural** | **Urban** | **OR** | **p** |
| --- | --- | --- | --- | --- |
| **Gender *** |  |  |  |  |
| Male | 170 | 762 | 1 |  |
| Female | 70 | 569 | 0.5 (0.4-0.7) | <0.001 |
| **Age** |  |  |  |  |
| Pup + Young | 51 | 251 | 1 |  |
| Adult + Old | 212 | 1157 | 0.9 (0.6-1.2) | 0.5 |
| **Body condition** |  |  |  |  |
| Good | 143 | 955 | 1 |  |
| Poor + fair | 120 | 453 | 1.8 (1.3 - 2.3) | <0.001 |
| **Activity^** |  |  |  |  |
| Not active | 354 | 1581 | 1 |  |
| Active | 263 | 1884 | 0.6 (0.5 - 0.7) | <0.001 |
| **Proximity to garbage (≤ 20 m)^** |  |  |  |  |
| No | 469 | 1787 | 1 |  |
| Yes | 148 | 1678 | 0.3 (0.2 - 0.4) | <0.001 |

*Gender of 23 FRD in rural and 77 FRD in urban areas could not be identified and were not included; ^Based on number of sightings, i.e. 617 for rural and 3465 for urban areas, respectively.

Supplementary table S4. The characteristics (gender, age, body condition and reproductive status) of free-roaming dogs in the different localities of the urban survey (Municipal Corporation, Panchkula) presented as odd ratios (OR) and their 95% confidence interval (CI).

|  | **Gender** | | | | **Age** | | | | **Body condition** | | |  | **Whether de-sexed?** | | | |
| --- | --- | --- | --- | --- | --- | --- | --- | --- | --- | --- | --- | --- | --- | --- | --- | --- |
| **Type of locality** | **Male** | **Female** | **OR (95%CI)** | **p value** | **Adult**  **+ Old** | **Young**  **+pups** | **OR (95%CI)** | **p value** | **good** | **poor**  **+ fair** | **OR (95%CI)** | **p value** | **Yes** | **No** | **OR (95%CI)** | **p value** |
| Residential | 311 | 241 | referent |  | 492 | 104 | referent |  | 406 | 181 | referent |  | 178 | 418 | referent |  |
| Industrial | 160 | 123 | 1.0 (0.7-1.3) | 0.9 | 263 | 49 | 1.1 (0.7-1.6) | 0.5 | 166 | 140 | 0.5 (0.4-0.7) | **<0.001** | 70 | 242 | 0.7 (0.5-0.9) | **0.01** |
| Res+Adm* | 51 | 32 | 1.2 (0.8-1.9) | 0.4 | 71 | 16 | 0.9 (0.5-1.7) | 0.8 | 63 | 19 | 1.5 (0.8-2.5) | 0.8 | 10 | 75 | 0.3 (0.1-0.6) | **<0.001** |
| Urban village | 99 | 62 | 1.2 (0.8-1.8) | 0.2 | 103 | 72 | 0.3 (0.2-0.4) | **<0.001** | 129 | 30 | 1.9 (1.2-3.0) | **<0.001** | 46 | 115 | 0.9 (0.6-1.4) | 0.7 |
| Admin# | 82 | 66 | 0.9 (0.6-1.4) | 0.8 | 134 | 14 | 2.0 (1.1-3.7) | **0.01** | 123 | 19 | 2.8 (1.7-4.9) | **0.01** | 11 | 137 | 0.2 (0.1-0.3) | **<0.001** |
| Perimeter | 22 | 15 | 1.1 (0.6-2.2) | 0.7 | 34 | 5 | 1.4 (0.6-4.2) | 0.5 | 29 | 8 | 1.6 (0.7-3.8) | 0.5 | 6 | 31 | 0.5 (0.2-1.1) | 0.07 |
| Unorganised** | 37 | 29 | 0.9 (0.6-1.6) | 0.9 | 61 | 8 | 1.5 (0.8-3.7) | 0.2 | 39 | 25 | 0.7 (0.4-1.2) | 0.2 | 20 | 49 | 0.9 (0.5-1.6) | 0.8 |

* Sectors comprising some residential and some administrative buildings. # Administrative areas. **The area comprised of Budhanpur, Indira colony and Rajiv colony which included unorganised houses and slums.
